# Reliability of the accelerometer to control the effects of physical activity in older adults

**DOI:** 10.1371/journal.pone.0274442

**Published:** 2022-09-12

**Authors:** Manne Godhe, Marjan Pontén, Johnny Nilsson, Lena V. Kallings, Eva A. Andersson

**Affiliations:** 1 Department of Molecular Medicine and Surgery, Karolinska Institutet, Stockholm, Sweden; 2 Department of Physical Activity and Health, The Swedish School of Sport and Health Sciences, Stockholm, Sweden; 3 Department of Neuroscience, Karolinska Institutet, Stockholm, Sweden; University of Bourgogne France Comté, FRANCE

## Abstract

**Background:**

Reliable physical activity measurements in community-dwelling older adults are important to determine effects of targeted health promotion interventions. Many exercise interventions aim to improve time spent sedentary (SED), in light-intensity-physical-activity (LPA) and moderate-to-vigorous-intensity-physical-activity (MVPA), since these parameters have independently proposed associations with health and longevity. However, many previous studies rely on self-reports which have lower validity compared to accelerometer measured physical activity patterns. In addition, separating intervention-effects from reactivity measurements requires sufficient test-retest reliability for accelerometer assessments, which is lacking in older adults.

**Objectives:**

The study objective was to investigate the reliability of sensor-based PA-patterns in community-dwelling older adults. Furthermore, to investigate change over time of physical activity patterns and examine any compensatory-effect from the eight-week supervised exercise-intervention.

**Methods:**

An exercise-group (n = 78, age-range:65-91yrs) performed two 1h-exercise sessions/week during eight-weeks. PA-pattern was assessed (using hip-worn accelerometers), twice before and once during the last-week of the intervention. A control-group (n = 43, age-range:65-88yrs) performed one pre-test and the end-test with no exercise-intervention. A dependent-t-test, mean-difference (95%-CI), limits-of-agreement and intraclass-correlation-coefficient-ICC were used between the two pre-tests. Repeated-measures-ANOVA were used to analyze any intervention-effects.

**Results:**

The exercise-groups´ two pre-tests showed generally no systematic change in any PA- or SED-parameter (ICC ranged 0.75–0.90). Compared to the control group, the exercise intervention significantly (time x group-interaction, p<0.05) increased total-PA-cpm (exercise-group/control-group +17%/+7%) and MVPA-min/week (+41/-2min) and decreased %-of-wear-time for SED-total (-4.7%/-2.7%) and SED-bouts (-5.7%/-1.8%), and SED-bouts min/d (-46/-16min). At baseline level, no significant differences were found between the two groups for any parameter.

**Conclusions:**

The current study presents a good test-retest-reliability of sensor-based-one-week-assessed-PA-pattern in older-adults. Participating in an 8-week supervised exercise intervention improved some physical activity and sedentary parameters compared to the control group. No compensatory-effect was noted in the intervention-group i.e., no decrease in any PA-parameter or increase in SED at End-test (in %-of-wear-time, min/day or total-PA).

## Introduction

Regular physical activity (PA) and reduce of sedentary behavior (SED) are associated with reduced risk of diseases, increased longevity, improved physical, mental and social health as well as cognition in older adults [1–7]. Mortality increases with self-reported SED-time over 7.5 h/d, with a highly increased risk above 10 h/d, while high levels of moderate-to-vigorous PA (MVPA) do not fully mitigate mortality risks associated with prolonged television watching [8,9]. A lower self-reported SED-time among adults including older adults is also linked with lower mortality [4,10], less frailty and better life quality, cognition and mental health [11].

Older adults (≥65 years) are the least physically active of any age group [12]. For this reason, ways to successfully promote PA are valuable in this age group [13,14]. To properly evaluate such interventions, better knowledge is needed regarding basic measurement characteristics, such as test-retest reliability of data from motions sensors, such as accelerometers. Measuring PA is associated with certain challenges: a valid instrument must capture its multi-dimensional nature (frequency, duration and intensity levels). The most commonly used method when measuring PA- and SED-behaviors in older adults is subjective questionnaires [15], which have limitations regarding validity and reliability [16,17]. To better understand how regular exercise and daily movement patterns are associated, it is necessary to objectively quantify all intensities of PA, and SED-behavior [18], preferably using motion sensors. PA- and SED-measurement by body-worn accelerometers is a fairly feasible method with reasonable validity [15]. Older adults are more often physically active in the lower-intensity activities, and more rarely activities of moderate-to-vigorous intensity measured with accelerometers [12]. It has previously been reported on how many days required for obtaining proper PA- and SED-accelerometer-assessed information, and if week-end days should be included or not when measuring older adults [19–21]. There is however, to our knowledge, a lack of studies on relatively healthy community-dwelling older adults evaluating the test-retest reliability from separate test occasions in close proximity to each other. Also, information regarding any possible compensatory effects in accelerometer-measured PA patterns during a supervised exercise intervention is lacking (see below).

Previous studies in older adults measured with accelerometers have often used different behavior-based methods, for example counseling and/or home-based methods such as repeated in-person or telephone counselling or exercise programs on DVD to promote more PA [22–24]. Some, but not all, of these authors report improvements in PA and/or SED behavior, although they have generally not included a control group. According to a recent meta-analysis, pedometer use does not increase PA in older adults [25]. Interventions with supervised physical exercise targeting relatively healthy community-dwelling older adults and seeking to increase PA- or decrease SED-behavior measured with accelerometers are lacking [15].

Moreover, to the best of our knowledge, no previous study on older adults has investigated various intensities of PA and SED-behaviors with accelerometers during two separate pre-test measurements in close proximity to each other, as intended in this study. It is valuable to investigate if major changes are seen for various accelerometer parameters between two separate pre-tests that may interfere on intervention outcomes when only one pre-test is utilized.

Also, previous physical exercise interventions with accelerometer measures are lacking regarding revealing many PA and SED parameters simultaneously, and further, an absence of presenting such data both in absolute time (minutes) and in percentage values of wear-time. The results may vary depending on whether the data is expressed in absolute or relative percentage values. In addition, supervised exercise interventions for relatively healthy community dwelling older adults have previously evaluated PA and SED parameters with self-assessed questionnaires, while such assessments with accelerometers are generally lacking or very scarce. Likewise, sufficient reliability is needed, to separate intervention-effects from measuring reactivity (i.e., increased activity levels for merely being included in a scientific study). As declared, test-retest reliability measures for accelerometer assessments in older adults is absent. Our intention in this study was to include the above-mentioned knowledge gaps in order to contribute novel and relevant data that might impact the health benefits of physical exercise in older adults.

The objective of the study was to determine test-retest reliability of accelerometer measured physical activity in older adults, more specifically regarding time spent at different activity intensities (total-PA, MVPA, LPA and Freedson-bouts) and time spent sedentary (SED-total and SED-bouts), i.e., daily movement patterns. The purpose was also to investigate change over time in daily movement patterns, measured with accelerometry, if there were any compensatory effect in PA pattern (i.e., decreases in PA levels or increases in time spent SED) following the participation in an eight-week supervised exercise intervention.

## Material and methods

### Study population

The study was designed as an eight-week supervised exercise intervention for community-dwelling healthy older adults with pre- and post-accelerometer-measurements. All participants were informed about the study and gave oral and written consent to participate in it. The study was approved by the regional ethics committee Stockholm, Sweden (ID:2017/2064-32).

The exercise group consisted of 78 older adults (70.9 ± 4.7 yrs, 65–91 yrs, 61.5% women, Table 1). The participants lived in a suburban community just outside Stockholm, and were recruited via advertising (in local media, social media and gathering locations for seniors) to this free of charge health project with supervised exercise organized by the Swedish School of Sport and Health Sciences and Solna municipality. The eight-week exercise intervention consisted of supervised physical group activities (60 minutes, twice weekly, for subgroups consisting between 10–30 participants), on Mondays and Wednesdays during the spring months March and April. All sessions started with 5–10 minutes of warm-up. The participants performed various exercises to music, such as aerobic gymnastics and muscle strengthening circuit training (exercise that was organized in stations with specific exercises, where participants switched between stations in a systematic manner) designed to activate all major muscle groups (leg, hip, back, abdominal, shoulder and arm muscles). During the sessions, the purpose was to increase heart rate and pulmonary ventilation without participants feeling exhausted. Also, various balance exercises were included for about 5 minutes in each training session. At the end of the sessions, last 5–10 minutes, there was a cool down of the intensity ending with breathing and relaxation exercises.

**Table 1:** Age and anthropometrics for the exercise group (n = 78) and the control group (n = 43) with mean values ±SD for age, weight, height and BMI for each group and for men and women separately. Exercise group values are also presented for each 5-year age interval, men and women together.

**Exercise group**	**n**	**Age (years)**	**Weight (kg)**	**Height (m)**	BMI (kg/m^2^)
**All**	78	70.9 ±4.7	72.7 ±13.6	1.70 ±0.10	25.1 ±3.9
**Men**	30	70.3 ±4.1	75.3 ±13.7	1.73 ±0.10	25.3 ±4.2
**Women**	48	71.2 ±5.0	71.1 ±13.5	1.69 ±0.10	24.9 ±3.7
**65–69 yrs**	34	67.0 ±1.3	73.3 ±11.6	1.71 ±0.10	25.1 ±3.4
**70–74 yrs**	29	71.7 ±1.2	70.8 ±16.1	1.69 ±0.09	24.8 ±4.4
**75–80 yrs**	13	76.4 ±1.4	75.6 ±15.1	1.73 ±0.09	25.2 ±4.0
					
**Control group**	**n**	**Age (years)**	**Weight (kg)**	**Height (m)**	BMI (kg/m^2^)
**All**	43	73.8 ±6.5	76.5 ±15.4	1.67 ±0.09	27.2 ±4.2
**Men**	13	75.7 ±4.2	87.6 ±11.4	1.77 ±0.07	27.9 ±3.1
**Women**	30	73.0 ±7.1	71.7 ±14.6	1.63 ±0.06	26.8 ±4.6

Hip-worn accelerometer recordings were performed during two pre-tests with one week in between (Pre-1 and Pre-2, before start of the intervention period) and one end-test (End, during the last week of the eight-week exercise period). In the control group, 43 older adults (73.8 ± 6.5 yrs, 65–88 yrs, 69.8% women, Table 1), were randomly recruited for accelerometer recordings only, i.e., with no supervised physical exercise. These subjects were recruited by asking by-passers on the streets, shopping centers and gathering locations for seniors, all living in the same suburb just outside of Stockholm. For exclusion criteria see below. The recordings were made with corresponding time intervals in the same springtime period, with only one test before (Pre-1) and one measure at the end of the eight-week period (End). These community-dwelling older adults were similar to the group receiving supervised exercise, although the intervention was not a randomized controlled study. The participants in the control group were instructed to continue their usual daily behaviors as normal.

Inclusion criteria in the exercise group were relatively healthy older adults (65≤ yrs) who, via advertising in local media, announced their willingness to participate in a free health project with supervised physical activity twice a week for two months. In the control group, inclusion criteria were relatively healthy older adults (65≤ yrs) asked on the streets who agreed to participate and perform measurements with accelerometers at the corresponding time-period and season as the exercise group. Exclusion criteria for both groups (recruited from the same suburb just outside central of Stockholm) were severe sickness for example due to heart failure or chronic obstructive pulmonary disease, and severe joint disease diagnosed by a medical doctor. Adherence of the older adults to the supervised physical activity program was generally 80%, i.e., the subjects could not participate for about 20% of all offered training sessions, due to various reasons, for example illness or a travel.

Body weight, length and age were self-rated in a short form at a local senior center just before the start of the assessment period, just after they were informed about the study and gave their oral and written consent to participate.

In the exercise group, 97 older adults were initially included for accelerometer measures of two pre-tests and the end-test. However, there was a sample loss of 19 participants who did not fulfill the participation in the study, due to illness, travel or that they did not successfully perform the minimum limit of four accelerometer wear-days. Thus, a total of 78 participants were included in analyzes in the current study for the exercise group. In the control group, 63 older adults were initially included. However, the sample loss was 20 participants who did not have a successful end-test due to illness, travel or that they did not wear the accelerometer for at least four days. Thus, a total of 43 participants were included in the analyzes in the current study for the control group.

### Accelerometer recordings and analyses

The accelerometer used was the GT3X (ActiGraph LCC, Pensacola, FL, USA). The ActiGraph accelerometer is a small (3.8 x 3.7 x 1.8 cm), lightweight (27 g) electronic device recording the acceleration of the participant´s movement. Subsequently, it provides a record of the intensity, frequency and duration of PA and SED behavior, summarized in units called counts. At the first meeting at the local senior center the participants received their accelerometer with oral and short written information about how to wear the sensor for at least one week. Then a suitable date was agreed upon about a little over a week ahead for the participant to return the accelerometer to the test leaders at the same local senior center. In the same way, it was agreed upon new dates for distribution and return of the accelerometer at the second pre-test (only for the exercise group) and at the end-test (both groups). The participants were instructed at each measurement to wear the accelerometer during all waking hours for at least seven consecutive days including weekend-days, although not during water-based activities. The accelerometer worn with an elastic belt, was placed over the participants´ right hip by the test leader on the first day of recording and was returned to the test leader approximately a week later. The accelerometers were prepared and initialized using ActiLife v.6.10.1 program, which was also used to download and process the collected data. The data were extracted as 60-sec epochs using a proprietary low frequency extension filter. Non-wear time was defined as at least 60 consecutive minutes with zero counts per minute (cpm). Participants included in the analyses wore the accelerometer for at least 4 days and 10 hours/day [19]. The raw recorded data from the accelerometer (sample rate 30 Hz) were analyzed regarding accelerations in the vertical direction (axis) for SED-behavior and PA at various intensities. Data were expressed as mean minutes spent in each intensity per day (min/day) as well as in percent of wear time in each intensity/total wear time (%-of-wear-time).

The following parameters were analyzed in absolute minutes and percentage of wear-time regarding the vertical axis acceleration. Total sedentary behavior (SED-total) was set as all time spent in 0–99 counts/min (cpm) [26–28]. The SED-bout was defined as spending ≥10 min within the range of 0–99 cpm [27]; light-intensity PA (LPA) was at 100–2019 cpm [12,26,28,29] and level for moderate-to-vigorous-PA (MVPA) was defined as ≥2020 cpm, vigorous being >5999 cpm [12,28–30]. The Freedson-bout (MVPA-bout) was defined as a consecutive period of >1952 cpm ≥10 min with a drop time of two minutes [12,30]. Also, daily cpm for the vector magnitude (VM) was analyzed [29,30]. Participants´ number of days with Freedson bouts ≥10 min for a total of at least 30 min was calculated to determine sufficiency for recommendations (at least 5 of 7 days i.e., 71.4% of measurement days) [31].

### Statistical analysis

Statistical calculations were performed using Statistica 13.5, (TIBCO Software Inc, Paulo Alto, California, US) and SPSS Statistics 26.0 Software package (SPSS Inc Chicago, Ill, US). Data are reported as mean and standard deviations (±SD). The data were normally distributed (Kolmogorov-Smirnov). For the two pre-tests a dependent t-test, mean differences with 95% confidence interval (95% CI), limits of agreement (LoA) and intraclass correlation coefficient (ICC) were calculated. Repeated measures-(RM)-ANOVAs were applied to assess changes between Pre-1- and End-test data for the exercise group and the control group. When significant time x group interactions were found, Fisher LSD post-hoc tests were applied to detect significant differences for each PA intensity and SED parameter. The effect size of the interaction effect between the exercise group and the control group over time (Pre-1-to-End) in the RM-ANOVA was analyzed using partial eta-squared (where 0.01 indicates a small effect, 0.06 a moderate effect and 0.14 a large effect). To detect significant differences between the sexes and also between the three age groups in the three test occasions for the exercise group, RM-ANOVA, with the Fisher LSD post-hoc-test was used. Significant level was set at p<0.05 for all analyses. Adjustment, with used significant level, was made for multiple testing between the sexes (p<0.01) and between the three age groups (p<0.0025), for proportional comparison.

## Results

**Fig 1:**
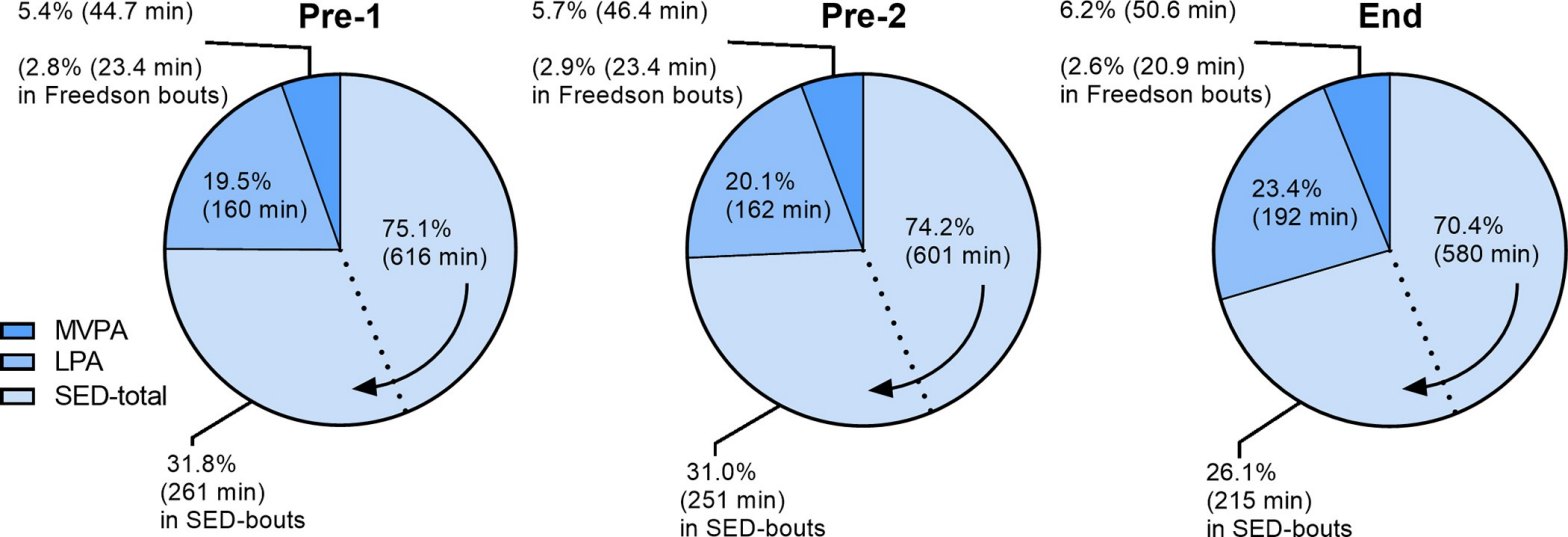
Results from the three test occasions Pre-1, Pre-2 and End in the exercise group. The average amount of time per day as well as percent of wear-time illustrated for SED-total, SED-bouts, LPA, MVPA and Freedson-bouts. The arrow indicates that the area for the SED-total also includes the area for the SED-bouts.

Fig 1 illustrates amount of time (in %-wear-time and min/d) for the accelerometer parameters SED-total, SED-bouts, LPA, MVPA and Freedson-bouts (MVPA-bouts) at the three test occasions Pre-1, Pre-2 and End-test in the exercise group.

## Test-retest reliability

We found no significant difference between the two pre-tests for any of the parameters SED-total, SED-bouts, LPA, MVPA or time in Freedson-bouts (in either min/day or %-wear-time), except for SED-total (only in %-wear-time, -1.2%, p = 0.018) and for total-PA (VM-cpm, +4.6%, p = 0.007), according to a t-test (Tables 2 and 3). Between Pre-1-test and Pre-2-test, the intraclass correlation coefficients (ICC) were generally good (in min/day and %-wear-time, see Table 2), and also for total-PA as VM-cpm: ICC = 0.91 (95% CI 0.86–0.95). Individually, assessed as limits of agreement (LoA), spontaneous changes in SED-total and MVPA of -6.7% to 5.1% (-142 to 113 min/d) and -3.0% to 3.5% (-25.5 to 28.9 min/day), respectively, were noted (Table 2). At a group level, assessed as mean difference (and its 95% CI), corresponding SED-total values were 0.78% (0.15% to 1.51%) and 14.64 min/d (-0.02 to 29.28 min/d) and MVPA values -0.28% (-0.67% to 0.08%) and -1.66 min/d (-4.78 to 1.78 min/day), respectively (Table 2). For further comparisons of the parameters, see Table 2. For VM-cpm, the mean difference (Δ 1–2) of 26.7 cpm, 95% CI (7.4 to 45.9 cpm) and LoA -140.6 to 194.0 cpm were noted.

**Table 2:** The exercise group Pre-1-test and Pre-2-test mean values, mean difference Pre1-Pre-2 (Δ1–2), 95% CI, limits of agreement (LoA) and intraclass correlation coefficient, ICC (95% CI) for SED-total, SED-bouts, LPA, MVPA and Freedson bouts in min/day and in % of wear-time. Significant differences between the two pre-tests (p<0.05), is marked with an asterisk (*) in the table.

				CI of difference		LoA		
***min/day***	**Pre-1**	**Pre-2**	**Δ 1–2**	**Lower 95**	**Upper 95**	***Lower 95***	***Upper 95***	**ICC (95%CI)**
**SED-total**	615.9	601.2	14.64	-0.02	29.28	*-142*.*0*	*112*.*7*	0.75 (0.61–0.84)
**SED-bouts**	261.1	250.6	10.49	-3.60	24.54	*-132*.*8*	*111*.*8*	0.83 (0.74–0.89)
**LPA**	159.8	161.7	-1.85	-7.22	3.51	*-44*.*8*	*48*.*5*	0.86 (0.79–0.91)
**MVPA**	44.7	46.4	-1.66	-4.78	1.78	*-25*.*5*	*28*.*9*	0.90 (0.84–0.93)
**Freedson-bouts**	23.4	23.4	0.00	-3.19	3.18	*-27*.*7*	*27*.*7*	0.82 (0.72–0.89)
***% of wear time***	**Pre-1**	**Pre-2**	**Δ 1–2**	**Lower 95**	**Upper 95**	***Lower 95***	***Upper 95***	**ICC (95%CI)**
**SED-total**	75.1%	74.2%*	0.78%	0.15%	1.51%	*-6*.*7%*	*5*.*1%*	0.90 (0.84–0.94)
**SED-bouts**	31.8%	31.0%	0.86%	-0.49%	2.29%	*-13*.*0%*	*11*.*2%*	0.89 (0.82–0.93)
**LPA**	19.5%	20.1%	-0.50%	-1.13%	0.06%	*-4*.*6%*	*5*.*7%*	0.89 (0.82–0.93)
**MVPA**	5.4%	5.7%	-0.28%	-0.67%	0.08%	*-3*.*0%*	*3*.*5%*	0.90 (0.84–0.94)
**Freedson-bouts**	2.8%	2.9%	-0.04%	-0.44%	0.33%	*-3*.*3%*	*3*.*4%*	0.82 (0.72–0.89)

**Table 3:** Mean values (±SD) in *min/day* (A-above) and in *%-of-wear-time* (B-below) for the exercise group (Exerc) and the control group (Contr) for various accelerometer parameters. A significant difference (*p1* < 0.05) between the two groups (seen only in End) is marked with the symbol ^**ϴ**^; and between End and either of the two pre-tests (marked at each pre-test) with a star (*) both for the exercise group and the control group. The control group performed only one pre-test. The effect size (ES) of the interaction effect comparing the exercise group versus the control group over time (Pre-1-to-End), measured with partial eta-squared, with significance level (*p2-*value), are presented below the End-tests´ average values.

A. Minutes	Pre-1 (Exerc / Contr)	Pre-2 (Exerc / Contr)	End (Exerc / Contr)
**SED-total**	615.9 (67.6)* / 631.4 (74.1)*	601.2 (78.6)* / -	579.6 (80.7) / 598.7 (93.8) *0*.*014*, *p2 = 0*.*190*
**SED-bouts**	261.1 (80.6)* / 282.4 (96.7)	250.6 (85.6)* / -	215.4 (88.8) / 264.4 (102.0) ^ϴ^ *0*.*026*, *p2 = 0*.*041*
**LPA**	159.8 (35.3)* / 162.2 (50.1)*	161.7 (33.2)* / -	192.3 (48.0) / 178.4 (53.0) *0*.*004*, *p2 = 0*.*477*
**MVPA**	44.7 (23.6)* / 37.6 (27.5)	46.4 (21.6)* / -	50.6 (23.2) / 37.3 (27.2) ^ϴ^ *0*.*042*, *p2 = 0*.*024*
**Freedson-bouts**	23.4 (18.9) / 20.7 (20.5)	23.4 (17.1) / -	20.9 (15.9) / 14.1*^↓^ (16.1) *0*.*024*, *p2 = 0*.*088*

**B. % wear-time**	**Pre-1 (Exerc / Contr)**	**Pre-2 (Exerc / Contr)**	**End (Exerc / Contr)**
**SED-total**	75.1 (5.3)* / 76.0 (7.0)*	74.2 (5.0)* / -	70.4 (6.7) / 73.3 (7.2) ^ϴ^ *0*.*023*, *p2 = 0*.*096*
**SED-bouts**	31.8 (9.7)* / 34.2 (11.9)	31.0 (9.7)* / -	26.1 (10.4) / 32.4 (11.3) ^ϴ^ *0*.*042*, *p2 = 0*.*025*
**LPA**	19.5 (4.3)* / 19.5 (5.7)*	20.1 (4.1)* / -	23.4 (5.6) / 21.8 (6.3) *0*.*005*, *p2 = 0*.*446*
**MVPA**	5.4 (2.8)* / 4.6 (3.4)	5.7 (2.7) / -	6.2 (2.9) / 5.0 (3.3) *0*.*039*, *p2 = 0*.*029*
**Freedson-bouts**	2.8 (2.3) / 2.4 (2.5)	2.9 (2.1) / -	2.6 (2.0) / 1.9*^↓^ (2.0) *0*.*024*, *p2 = 0*.*093*

### Change over time

#### Pre-1-test vs End-test

**Fig 2:**
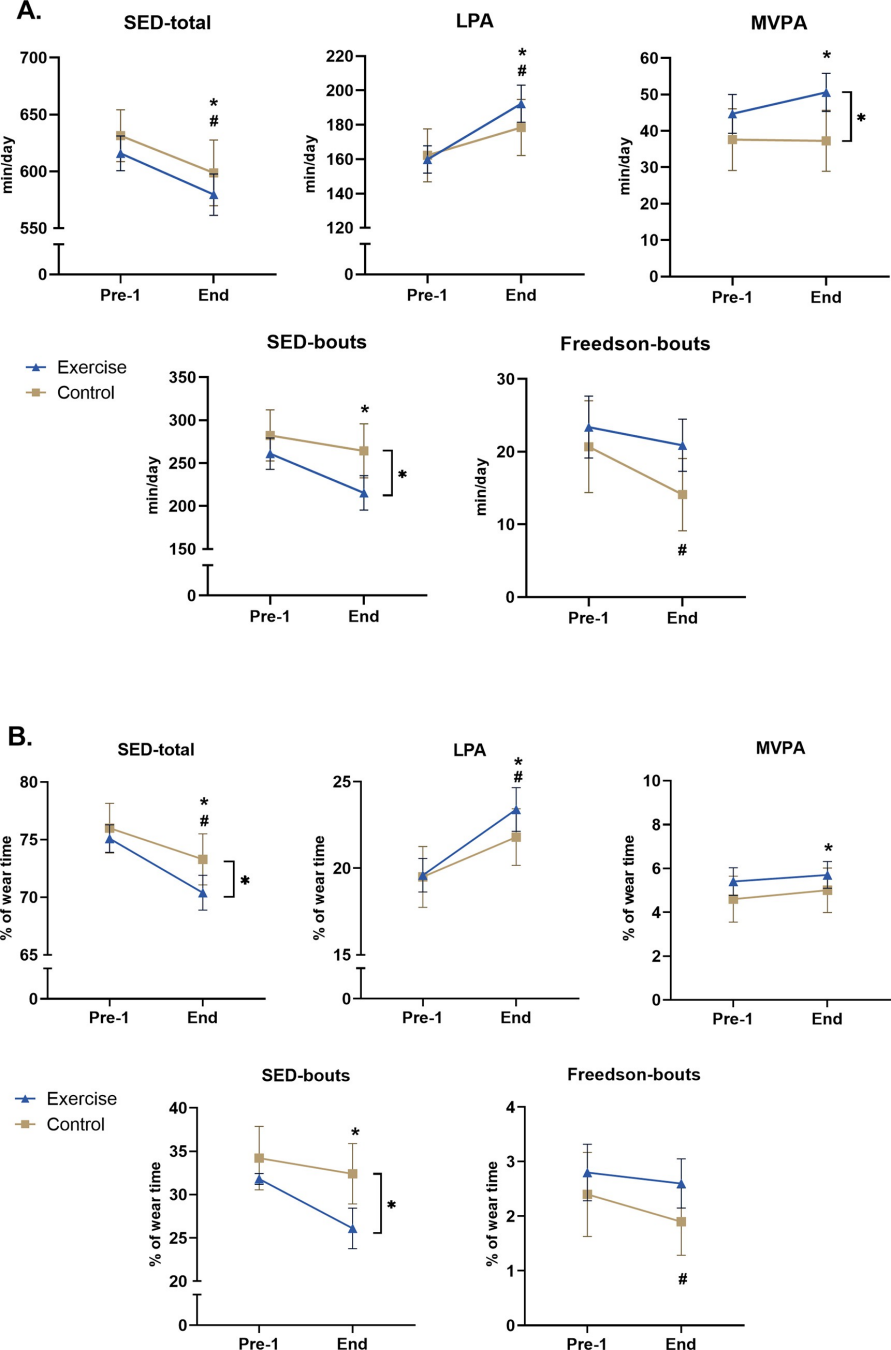
Average values (with 95% CI) in *min/day* (**A**-above) and in *%-of-wear-time* (**B**-below) for the exercise and the control groups for various accelerometer parameters. A significant difference (p < 0.05) between the two groups (only seen at End) is noted with] *, and between Pre-1-test and End-test with * for the exercise group, and **#** for the control group.

The exercise group showed a significant daily decrease of 36.3 min (4.7% of total-daily-wear-time) in SED-total, and 45.7 min (5.7%) of SED-bouts. In parallel, the exercise group showed a significant increase of 32.5 min (3.9%) in LPA, and 5.9 min (0.8%) in MVPA (Fig 1, Table 2 and Fig 2). No significant improvement was observed for Freedson-bout. Total-PA values as vector magnitude (VM) cpm were 575.5, 602.2 and 675.2 for Pre-1, Pre-2 and End-tests respectively, with a significant improvement between each pre-test compared to End-test (+17.3% and +12.1%). All described improvements were significant at the level p<0.01.

In the control group, a small but significant improvement was found between Pre-1-test and End-test for the parameters SED-total (-2.7%) and LPA (+2.3%) in both min/d and %-of-daily-wear-time (all p<0.01, Table 3B) and for VM-cpm (538.4. vs 574.5 cpm, +6.7%, p = 0.04).

In *Pre-1-test*, no significant difference in min/d or %-wear-time was seen between the exercise and the control group in any of the measured parameter SED-tot, SED-bouts (minimum of consecutive 10 min periods in sedentary counts), LPA, MVPA, Freedson-bouts or total-PA as VM-cpm (Fig 2 and Table 3).

When employing a RM-ANOVA, significant terms were noted regarding an interaction effect for VM-cpm (F = 8.303, df = 1, p = 0.005), MVPA-min/d (F = 4.277, df = 1, p = 0.041), SED-bouts-min/d (F = 3.396, df = 1, p = 0.0496), SED-% (F = 5.730, df = 1, p = 0.018), SED-bouts-% (F = 5.826, df = 1, p = 0.017). Post-hoc analyses revealed significant differences post-intervention between the exercise group compared to the control group for total-PA (as VM cpm, p = 0.005), MVPA (min/d, p = 0.006), SED-bouts (min/d, p = 0.005), SED-total (for % wear-time, p = 0.018) and SED-bouts (% wear-time, p = 0.003). In further analyzes, the effect size (ES, measured via partial eta-squared) was significant, though small, comparing the exercise group versus the control group over time (Pre-1-to-End), for total-PA as VM-cpm (*0*.*034*, *p2 = 0*.*043*), MVPA-min/d, SED-bouts-min/d, MVPA-% and SED-bouts-% (ES-values shown in Table 3).

The amount of time spent in vigorous intensity was very low (varying between 0.24–1.14 min/day) on all test occasions for both groups.

### Compensatory behavior

Based on within-group analyses in the exercise group, we found no evidence for compensatory behaviors at the End-test with decrease in any measured PA-parameter or increase in time spent sedentary. Thus, the significant improvements of several parameters found for the exercise group did not result in a significant undesirable change of any other assessed PA- or SED-parameter.

### Sex and age group comparisons in the exercise group

At both pre-tests the values for women, compared to men, tended to be lower for SED-behavior and higher for LPA and total-PA (VM-cpm), although with no significant difference between the sexes, neither seen at the End-test (Table 4). From Pre-1-test to End-test and from Pre-2-test to End-test a significant improvement was generally seen for both sexes regarding SED-total, SED-bouts, LPA and VM-cpm and among women also for MVPA, (p1<0.01). The values for VM-cpm for men/women, in the three tests Pre-1, Pre-2 and End were 536/602, 566/628 and 632/702, respectively, with no significant difference between the two pre-tests for any sex. A moderate to large effect size (ES) was seen between men versus women over time (Pre-1-to-End) for LPA and SED-bouts in both units (min/d and %-wear-time) and also for SED-total expressed in %-wear-time (assessed with partial eta -squared, Table 4). No sex differences (via partial eta-squared) were seen over time for MVPA and Freedson-bouts, neither for total-PA (VM-cpm Pre-1 vs End 0.036, p2 = 0.094, Pre-2 vs End 0.037, p2 = 0.090.

**Table 4:** Exercise group average values for men and women (±SD) in *min/day* (A-above) and in *%-of-wear-time* (B-below) for various accelerometer parameters. A significant difference (*p1*<0.01) between End and either of the two pre-tests is marked at each pre-test with an asterisk (*) for both sexes. The effect size (ES) of the interaction effect comparing men versus women over time, measured with partial eta-squared with significance level (*p2-*value) are presented below the average values for i) Pre-1 (for changes from Pre-1-to-End), and ii) Pre-2 (for changes from Pre-2-to-End).

**A. Minutes**	**Pre-1 (Men / Women)**	**Pre-2 (Men / Women)**	**End (Men / Women)**
**SED-total**	636.6 (73.8)* / 602.9 (60.4)* 0.*038*, *p2 = 0*.*086*	620.2 (99.1) / 589.3 (60.8) *0*.*028*, *p2 = 0*.*141*	590.9 (93.6) / 572.5 (71.6)
**SED-bouts**	308.5 (72.0)* / 231.4 (71.4)* 0.*168*, *p2 < 0*.*001*	286.6 (92.2)*/ 228.1 (73.7)* 0.*113*, *p2 = 0*.*003*	248.7 (89.3) / 194.6 (82.8)
**LPA**	143.5 (40.2)* / 170.0 (27.2)* 0.*060*, *p2 = 0*.*031*	150.9 (36.2)*/ 168.4 (31.1)* 0.*037*, *p2 = 0*.*091*	185.2 (54.1) / 196.8 (43.9)
**MVPA**	44.5 (24.1) / 44.9 (23.6)* 0.*006*, *p2 = 0*.*518*	44.1(20.1) / 47.8 (22.6) *0*.*014*, *p2 = 0*.*307*	46.7 (20.6) / 53.0 (24.5)
**Freedson-bouts**	24.7 (21.8) / 22.5 (17.0) *0*.*002*, *p2 = 0*.*689*	22.6 (17.5) / 23.9 (17.1) *0*.*011*, *p2 = 0*.*365*	17.8 (13.6) / 22.9 (17.1)
			
**B. % wear-time**	**Pre-1 (Men / Women)**	**Pre-2 (Men / Women)**	**End (Men / Women)**
**SED-total**	77.2 (5.9)* / 73.6 (4.5)* 0.*059*, *p2 = 0*.*032*	75.9 (5.4)* / 73.0 (4.4)* 0.*048*, *p2 = 0*.*054*	71.7 (8.0) / 71.7 (8.0)
**SED-bouts**	37.5 (9.0)* / 28.1 (8.4)* 0.*173*, *p2 < 0*.*001*	34.9 (9.3)* / 28.1 (9.1)* 0.*117*, *p2 = 0*.*002*	30.1 (10.5) / 23.2 (9.6)
**LPA**	17.4 (4.7)* / 20.9 (3.4)* 0.*070*, *p2 = 0*.*019*	18.6 (4.3)* / 21.1 (3.7)* 0.*042*, *p2 = 0*.*071*	22.5 (6.5) / 24.1 (4.9)
**MVPA**	5.4 (3.1) / 5.5 (2.7)* 0.*004*, *p2 = 0*.*581*	5.5 (2.6) / 5.9 (2.7) *0*.*011*, *p2 = 0*.*358*	5.7 (2.6) / 6.5 (3.0)
**Freedson-bouts**	3.0 (2.8) / 2.7 (2.0) *0*.*001*, *p2 = 0*.*654*	2.8 (2.2) / 3.0 (2.1) *0*.*009*, *p2 = 0*.*421*	2.2 (1.7) / 2.8 (2.1)

Between the *three age groups* (65-69/70-74/75-80 yrs), there were no significant differences for any parameter on any of the three test occasions (Table 5). Neither the two pre-tests showed any significant differences within each age group. A significant improvement was frequently seen for all three age groups regarding change over time from Pre-1-to-End and from Pre-2-to-End among the parameters total-PA (VM-cpm), LPA and for SED-bouts in one or both units, and for SED-total (in %-wear-time). The values for VM-cpm among the *three age groups* (65-69/70-74/75-80 yrs), were in Pre-1-test: 588/585/553, Pre-2-test: 632/580/598 and End-test: 702/647/680 respectively. No significant interaction effect over time was seen between any of the three age groups, measured with partial eta-squared (either from Pre-1-to-End or Pre-2-to End), for any of the analyzed accelerometer parameters.

**Table 5:** Age group (65–69 yrs, 70–74 yrs and 75–80 yrs) average values (±SD) in *min/day* (A-above) and in *%-of-wear-time* (B-below) for various accelerometer parameters in the exercise group. Significant difference (p < 0.0025) between End-test and either of the two pre-tests, within each age group is marked at each pre-test with *.

**A. Minutes**	**Pre-1 (65–69 yrs / 70–74 yrs / 75–80 yrs)**	**Pre-2 (65–69 yrs / 70–74 yrs / 75–80 yrs)**	**End (65–69 yrs / 70–74 yrs / 75–80 yrs)**
**SED-total**	620.9 (76.3)*/ 616.8 (63.7) / 601.2 (59.0)	599.9 (61.0) / 616.2 (99.0) / 580.3 (64.1)	568.9 (83.8) / 593.9 (69.3) / 597.7 (101.7)
**SED-bouts**	270.2 (82.9)*/ 251.6 (75.6) / 262.2 (94.2)	249.6 (71.2)* / 255.2 (93.0) / 251.0 (110.7)	212.4 (90.6) / 224.0 (66.2) / 213.4 (129.9)
**LPA**	163.2 (34.5)*/ 165.3 (31.5)* **/** 138.8 (41.8)*	166.4 (31.4)* / 163.6 (30.9)*/ 144.7 (40.8)*	196.6 (46.5) / 188.3 (42.6) / 191.9 (67.2)
**MVPA**	46.4 (17.1)*/ 44.8 (25.9) / 46.5 (30.2)	50.2 (17.0) / 43.4 (24.6) / 48.6 (22.3)	54.9 (20.0) **/** 46.4 (26.1) **/** 54.0 (21.0)
**Freedson-b.**	24.9 (16.9) / 22.5 (18.4) / 24.8 (24.7)	26.3 (16.0) / 20.3 (18.4) / 25.9 (16.1)	23.9 (15.6) **/** 18.7 (18.4) **/** 20.7 (9.0)
			
**B. % of wear-time**	**Pre-1 (65–69 yrs / 70–74 yrs / 75–80 yrs)**	**Pre-2 (65–69 yrs / 70–74 yrs / 75–80 yrs)**	**End (65–69 yrs / 70–74 yrs / 75–80 yrs)**
**SED-total**	74.6 (5.3)* / 74.6 (5.1)* / 76.6 (6.5)*	73.4 (4.5)* / 74.7 (5.4)* / 75.2 (6.5)*	69.3 (6.9) / 71.7 (5.0) / 70.2 (9.4)
**SED-bouts**	32.4 (9.3)* / 30.4 (9.6) / 33.9 (13.2)*	30.5 (8.4)* / 30.7 (9.6) / 32.9 (14.7)*	25.8 (10.9) / 27.1 (8.0) / 25.8 (15.0)
**LPA**	19.8 (4.4)* **/** 20.1 (4.0)* **/** 17.5 (4.9)*	20.4 (3.7)* / 20.0 (4.0)* / 18.7 (4.8)*	24.0 (6.9) / 22.7 (4.8) / 23.2 (7.8)
**MVPA**	5.6 (2.1)* / 5.4 (2.9) / 5.8 (3.8)	6.2 (2.2) / 5.3 (2.9) / 6.2 (2.6)	6.8 (2.6) / 5.6 (3.1) / 6.6 (2.8)
**Freedson-b.**	3.0 (2.0) / 2.7 (2.1) / 3.1 (3.2)	3.3 (2.0) / 2.5 (2.2) / 3.3 (2.0)	2.9 (1.9 / 2.3 (2.2) / 2.5 (1.2)

## Discussion

The current study found a good test-retest reliability of sensor-assessed physical activity and sedentary patterns in older adults. The exercise group compared to the control group significantly increased total-PA and MVPA (min/day), and decreased SED-total and SED-bouts (%-wear-time), and SED-bouts (min/day). At baseline-level, no significant differences were found between the two groups for any measured parameter. Thus, the current study suggests that the supervised exercise intervention in this population of community-dwelling older adults succeeded in improving several accelerometer-assessed daily-movement- and sedentary parameters without undesirable compensatory changes in PA- and SED-patterns.

To the best of our knowledge, this is the first study on older adults investigating accelerometer measurements in two separate pre-tests in close temporal proximity to each other (see also [20]). Between the Pre-1-test and Pre-2-test the intraclass correlation coefficient (ICC) varied here from 0.75 to 0.91 among all parameters. A review by Falck et al. (2016, [15]) pointed out that no previous study has conducted within-sample reliability among the objective measures used in older adult intervention. However, an earlier study, performing a retest one and two years after the initial test, showed ICCs ranged from 0.67–0.82 (for SED, LPA, MVPA, MVPA-bouts expressed in min/d and VM-daily counts), with hip accelerometers used in older women [32].

The *exercise group* showed significant improvements, with increase of total-PA (VM-cpm), LPA and MVPA, and decrease of SED-total and SED-bouts, from Pre-1-test to End-test. The reduction in SED behavior is mainly exchanged with LPA and to some extent MVPA. Even though most recommendations are related to MVPA, some studies also highlight the importance of LPA, especially as a substitution for SED behavior (see below).

MVPA increased by 5.9 min/day (i.e., 41 min/week at Pre-1-test to End-test) although the weekly 2h of physical activity sessions with combined aerobic and strength-training were designed for moderate-to-high intensities. An accelerometer cannot always measure the complexity of higher PA intensities correctly, especially during strength training tasks (see also below). However, no significant increase was seen in the control group for MVPA between Pre-1-test and End-test. Regarding the WHO recommendations of 150–300 min/week in MVPA (or at least 75–150 min/week in vigorous PA) [1,7,13], our findings show that sufficient mean daily average time values exceeded 150 minutes MVPA weekly in both groups on all test occasions (Pre-1, Pre-2 and End). Yet, when applying the Freedson-bout criterion (minimum 10 min bouts MVPA), only exercise group Pre-tests 1 and 2 had sufficient time in MVPA, and a small decrease in the End-test resulted in insufficient time in MVPA-Freedson-bouts. Nevertheless, when counting number of days on which each person reached 30 min of MVPA in Freedson-bouts at least five days/week, at most 17.4% reached this recommendation in the Pre-tests and at most 8.7% at the End-test in both groups. However, the previously emphasized accumulation of MVPA-time in Freedson-bouts of at least 10 min is no longer included in the new WHO recommendations [7]. For example, mortality risk reductions associated with MVPA are independent of how activity is accumulated [33]. Moreover, increased time spent in MVPA have positive associations with numerous health outcomes and increased longevity in several cross-sectional and longitudinal accelerometer studies (see below).

The amount of time spent in vigorous intensity was very low (about 7 min/week) on all test occasions for both groups. Very few previous studies have reported older adults´ vigorous intensity minutes alone; most report MVPA instead. Possibly due to the uncertainty of accelerometry measurements at higher intensities, especially in older adults (see below) [12,34].

The control group showed a significant increase in total-PA (VM-cpm) and LPA (min/d and % wear-time) and a significant decrease in SED-total (min/d and %-wear-time) from Pre-1-test to End-test. However, the changes were significantly greater in the exercise group than in the control group for total-PA (VM-cpm) and SED-total (in % wear time). This suggest that being measured with accelerometers alone may contribute to some PA and SED pattern improvements, as seen for the control group, and this should be considered in accelerometer interventions for older adults. A seasonal effect, measuring the end-test later in springtime, could possibly also have influenced the PA pattern improvements seen in the control and the exercise group.

### Exercise interventions in older adults

A great challenge remains when it comes to the implementation of evidence-based methods to increase PA- and reduce SED-behavior in older adults. Most previous exercise-interventions in older adults have been measured with self-reports (which have lower validity), rather than accelerometer measurements, and reliability measures performed with accelerometers are lacking (see above, [15]). Furthermore, there is a scarcity of previous accelerometer-assessed interventions for relatively healthy older adults receiving supervised physical exercise. In this context, to our knowledge, presented data are the first assessing and showing significant improvement in several accelerometer-assessed parameters for both SED and PA as well as total-PA among community dwelling older adults. Also, conducted with inclusion of two pre-tests and a control group for comparison. A significant improvement of *daily mean values* from Pre-1-test to End-test was noted for our exercise group regarding total-PA (VM-cpm +17%), MVPA (+6 min), LPA (+33 min) SED-total (-36 min) and SED-bouts (-46 min), but not for Freedson-bouts (MVPA-bouts).

Below we describe some findings from previous accelerometer interventions, including supervised regular physical exercise for older adults, also with a control group for comparison.

Three *supervised physical exercise interventions for elderly* found an *increase in PA* measuring one or two daily movement parameters (but not assessing SED-behavior: i) for MVPA (+39 min/week) only for those insufficiently active at baseline-level (i.e., < 150 min MVPA/week), but not among older adults in general (2 exercise-sessions/w for 10 weeks, n = 93, using arm accelerometers) [35]; ii) for LPA (+17 min/d) and MVPA (+7 min/d) via a multilevel PA intervention, including supervised group walks (3 months, 70% ≥ 80yrs, n = 300, using hip accelerometers) [36]; iii) for MVPA (although not reporting the amount of MVPA-change) with aerobic dance or aerobics (3 sessions/w, 6 months, n = 250, using hip accelerometers using a lower MVPA-cut-point ≥1041 cpm) [37].

Conversely, in some *supervised exercise interventions* (3–11 months) *no improvements* of different daily PA intensities and SED-time were observed using accelerometer assessments [38–41]. Moreover, total energy expenditure did not alter with endurance exercise (3 exercise-sessions/w for *8 or 14 weeks)* [42,43]. Furthermore, total-PA was unchanged (counts/d) during a *12-week*-intervention (2 sessions/w) [44]. In this study, the increase in moderate intensity during the isolated training-sessions was compensated by a decrease in “non-training-PA”. No such undesirable compensatory effect was noted in the exercise group in our study.

Among older adults with a *special disease* an improvement of total-PA (the only presented PA-intensity parameter) was reported (not for controls) but for i) *heart failure* patients with seated exercises (2 sessions/w for 3 months) [45]; ii) those with *type-II diabetes* with a partly supervised walking intervention (3 sessions/w, 6 months) [46]; iii) *institutionalized elders* with either aerobic or strength training (2 sessions/w, 1 year) [47]. iv) Finally, elderly with *obesity*, increased daily MVPA, LPA and step counts (with 18 min, 13 min and about 3000 steps, respectively) in a weight-loss-intervention, including diet and supervised treadmill walking exercise (4 sessions/w, 5 months) [48].

Thus, our study is the first to report that supervised exercise twice weekly for healthy community-dwelling older adults may improve several physical activity and sedentary parameters, without undesirable compensatory changes.

### Methodological considerations

There is some discrepancy between the exercise and the control group regarding mean age (70.9 vs 73.8 yrs), BMI (25.1 vs 27.2 kg/m2) and number of participants (78 vs 43). There were also somewhat fewer men than women, and fewer of the oldest age group versus the two younger age groups. These differences could possibly result from random selection of the controls versus volunteer participation in the exercise group. However, no significant differences in daily movement patterns were seen between the exercise group and control group at pre-tests, suggesting that there was no skewing between the groups at baseline-level.

We recorded SED-bouts in 10-minutes intervals, as in a previous cross-sectional study in older adults [27]. This interval is slightly smaller than in other cross-sectional investigations, which for example, recorded 30 min-SED-bouts in older adults [26] and 20 min-SED-bouts in middle-aged adults [29]. For middle-aged adults, taking several small SED-breaks every 20 minutes, compared to prolonged SED-time, show lower metabolic risks in overweight/obese [49].

Since both self-reported data and accelerometry have different methodological advantages and disadvantages, a combination of these two measures might be appropriate to get the clearest possible picture of an individual´s PA- and SED-behavior. For instance, accelerometers may not reflect the advantages of strength training or cycling, and further, they cannot generally be used in water exercises/swimming (see also above and below).

The validity of accelerometry has previously been studied in different ways. The accelerometer’s ability to predict energy expenditure compared to criterion methods, i.e., double labeled water or direct or indirect calorimetry has proven to be reasonable and seems to be independent on accelerometer make or model [50–53]. Instead, discrepancies between studies validating different accelerometers are more likely attributable to differences in use of epoch, cut-points, frequency filters and investigated intensities [54].

Previous studies have shown good validity when measuring low intensities and SED-behaviour. However, when measuring PA, validity is lower with increasing intensity, and especially low in vigorous intensity (>6 METs) [55]. In our study the exercise group, although receiving 60 min of supervised training sessions (2/w) of mainly moderate-to-high intensity physical activities, showed low values in time spent in vigorous intensities (7.5 min/week on average during the last week of the eight-week exercise period). A systematic review published in 2014 regarding accelerometer measurements in older adults concluded that there is no standardization regarding cut-points for PA or SED behaviour, but setting the cut-points too high or too low will markedly affect the results [56]. So far, no real consensus exists regarding cut-points for PA or SED behavior for older adults [57]. Cut-points for the ActiGraph accelerometer (GT3X) have previously been developed in laboratory settings for older adults [58]. However, in their study the epoch length was 1s whereas we used 60 s. These authors suggested the cut-point for 3 MET (MVPA cut point) at VM 2751 cpm, whereas we set the same cut-point to VM 2020 [12]. Another study found that postmenopausal women show good agreement for 150 min/w of MVPA with Troiano and Freedson cut-points (≥2020cpm and ≥1952cpm, respectively), but not with the cut-points used by Copeland & Esliger (≥1041cpm) or Sasaki (≥2691cpm) [30]. As described earlier, cut-points are highly dependent on epoch time and also whether the vertical axis or the vector magnitude (VM) is being used; the different approaches are not directly comparable [59]. One study investigated cut-points for SED behavior in older adults [60]. We used the limit <100 counts. However, our cut points for both SED-behaviour and PA-intensities (using the vertical axis acceleration) are in line with many previously used cut-points. In the present study we were interested to investigate possible changes in activity patterns during an intervention, therefore using the same cut-points and epoch on all test occasions was of importance. The cut points were set to reflect resting metabolic energy turnovers (METs) of 1.5–3 METs for LPA and >3 METs for MVPA. This is potentially problematic when studying older adults where the same energy turnovers is less likely to represent intensities of a whole adult population (from 18 years+). Further, a certain absolute MET-/cut-point limit (i.e., MVPA≥2020 cpm) may result in different relative intensities between various older adults depending on each individual´s fitness level. It is more likely that the cut points for older adults should be set lower for both moderate and vigorous PA due to age-related decline in fitness. However, in a test–retest design or an intervention design, studying differences, a comparison can still be made even though the cut points possibly could be unrepresentative of the true METs.

One study [19], investigated the number of days required for reliable data in older adults, comparing both the cut-points suggested by Aguilar-Farias et al. [60]) and the commonly used Freedson cut-points. They reported that for uniaxial accelerometer data 3.0 to 4.9 days are necessary for reliably estimating time spent in SED and PA in older adults when using widely known cut-points for uniaxial accelerometer data. They also stated that when using cut-points for VM of triaxial-accelerometer data, 2.5 to 4.5 days was necessary for achieving an ICC of 0.80. Further, there was no evident requirement to include at least one weekend day for reliably estimating PA-intensities and SED-behavior in older adults, as the authors did not observe a systematic difference in estimates between weekdays and weekend days. However, a review by Heesch et al.(2018) reported that weekend days should be included for proper accelerometer-measured SED and PA parameters in older adults [20]. Weekend days were included in our study.

#### Accelerometer measures related to previous reports

In what follows, our present data is compared to previous reports with older adults (with predominantly similar one-occasion accelerometer assessments, reported mostly in one unit, either min/d or %-wear time). *Our daily baseline values* were for time spent *in SED-tot*: higher [27,61–73], similar [66,74], and lower [75], *in SED-bouts*: lower [27]; *in LPA*: higher or similar [39,75,76], and lower [26,27,63,65,67,70,71,74]; *in MVPA*: higher [12,26,27,39,62,63,65,67–69,71,73,76,77], similar [61,70], and lower [74,75]; *in VIG*: higher [12], and similar [74]. Compared to middle-aged (50–64 yrs), our older adults showed higher levels in percent (%) for total daily SED-behavior, less in SED-bouts and LPA, but slightly higher or similar %-values for MVPA [31].

### Sex differences

Older women in the present study tended to spend more time in LPA and total-PA (VM-cpm), and less time sedentary (SED-total and SED-bouts) than older men did at baseline-level, though the differences were not significant. These results are partly in line with previous accelerometer reports of older women spending more time in LPA and less in SED behavior and MVPA than older men [12,28,64,65,68,69,75,78]. Moreover, there are reports of older women having slightly more MVPA-time than men [75]. Overall, PA volume has been reported similar between the sexes [12,68] or slightly higher for either older women [75] or older men [65]. Furthermore, among middle-aged adults (50-64yrs), men spent more time in SED and MVPA and less time in LPA than women, with no difference between the sexes regarding total-PA measured as mean cpm [31]. At the End-test we found no significant differences between the sexes for any investigated parameter.

### Age differences

We found no significant age group difference at baseline-level or at the End-test for any parameter (65-69/70-74/75-80 yrs). Previously, no significant age difference has been shown in one test occasion, for time in SED behavior, LPA, MVPA or total PA, between age groups 70–80, 60–70 and 49–59 yrs, except when comparing >80yrs with younger age groups, in the mentioned daily movement parameters [68]. Another study, assessing total PA solely, showed older participants (80–93 yrs) to be less active than younger participants (65–79 yrs), [79]. However, increased SED-time and decreased LPA- and MVPA-time with advancing age have also been seen for each 5-years-intervals from 70 to ≥85 yrs (with more marked changes ≥ 80 yrs) [73,75] and for a group of 60–75 yrs compared to an age group between 18–59 yrs [80]. It was likewise within different middle-aged groups for time in MVPA and total-PA (60–65 yrs having lower values than 50–59 yrs), but not for LPA or SED behavior [31].

#### Accelerometer measures related to health benefits in older adults

In this study significant improvements were noted in the exercise group at End-test in accelerometer-assessed parameters; total-PA (cpm), MVPA, LPA, SED-total and SED-bouts (all in min/d and %-of-wear-time). These improvements may contribute to improved health in various ways. For instance, links between *positive health outcomes* and time spent in SED, LPA, MVPA and total PA *for older adults* have been shown in several longitudinal and cross-sectional accelerometer reports, presented below:

Accelerometer investigations of longevity and improved health with *only longitudinal data* have shown that higher SED-time is related to higher mortality in less active middle-aged and older adults, where 30–40 min of MVPA/d attenuate the association between SED-time and risk of death [81]. In contrast, a meta-analysis including more than 1 million adult men and women showed that higher activity with 60–75 min MVPA/d, seem to eliminate the increased mortality risks linked with high total sitting time (>8h/d) [3]. Shortened SED-time and increased LPA- and MVPA-durations as objectively measured in elderly people is associated with better health and a longer life in longitudinal studies [28,71,72]. However, breaking up SED-time and accumulating MVPA (lasting ≥10 min) did not alter the relations with mortality here. Substituting 30 min of SED-time with LPA may reduce the risk of all-cause mortality by 11% and reduce the risk of cardiovascular disease by 24% according to another longitudinal study [28]. Further, replacing 30 min of accelerometer-assessed SED-time with LPA is associated with a 14% reduced mortality risk in a population of 50–85 years, shown with isotemporal substitution analyses [77]. Moreover, replacing 10 min SED-time with MVPA is associated with reduced CVD mortality risk [28]. Like MVPA, total-PA volume is also a strong predictor of mortality, this is in agreement with findings that LPA can give substantial survival benefits, highlighting the importance of total-PA and LPA in public health contexts for elderly people [71]. In the same study, it was shown that SED-time was an essential risk factor of all-cause mortality and CVD mortality independently of participants achieving the recommended dose of MVPA. Participants who spent almost 10 h/day sitting down had an over 2.5 times larger risk of death from any cause than those with only 6.5 sedentary h/day, and this association remained after adjusting for MVPA.

Accelerometer publications including summarized results from *both cross-sectional and longitudinal studies*, have reported that reduced SED-time in older adults is associated with longevity, better physical and cognitive functions, mental health, quality of life and reduced cardiometabolic diseases [82]. The authors also stated, in their review, that self-reported tools underestimated SED-time. The association between objectively measured SED and physical performance in the elderly is strong and further highlights the importance to reduce SED-time in order to improve physical function in older adults [83]. Separately accelerometer-assessed LPA-time and MVPA-time among older adults are associated with less chronic inflammation [39]. In addition, shortened SED-time and increased LPA- and MVPA-durations are associated with lower arterial stiffness [84].

Accelerometer studies with *cross-sectional data* solely have shown significant associations between MVPA-duration and several health benefits, body composition and walking-speed tests [74], as well as with mental and psychosocial health and health-related life-quality, physical fitness, lower aortic arteriosclerosis, osteoporosis, sarcopenia, and components of the metabolic syndrome, especially hypertension and hyperglycemia [64]. In addition, increased SED-time and fewer breaks are related to decreased physical function, independently of MVPA in older adults [69]. Furthermore, replacing 30 min of accelerometer-assessed SED-time with LPA is associated with better physical health and well-being in older adults [63]. These facts are of great clinical and public health importance, due to the potential of LPA to confer a higher compliance rate for regular PA [76]. Moreover, reallocating 30 min SED time in bouts or non-bouts to MVPA is related to lower waist circumference, lower BMI and higher HDL (high density lipoproteins) levels in older adults with prediabetes and type-II diabetes, and reallocating time in SED-bouts to LPA is linked to lower waist circumference, shown in isotemporal substitution analysis [70]. Such analysis also showed that replacement of 10-minutes MVPA with either LPA or SED is associated with increased clustered metabolic risk score and waist circumference among older women [27]. All associations between SED-time and metabolic risk outcomes were lost once variation in total accelerometer counts was adjusted for; thus, PA and not SED time per se influences clustered metabolic risk in this cross-sectional study. Furthermore, in older women (65–70 yrs), reallocating 30 min of SED time with LPA or MVPA is linked to reduced fibrinogen, and with MVPA alone to a lower C-reactive protein (CRP) [26]. CRP and fibrinogen are related to metabolic and cardiovascular disease (CVD) (partly via endothelial dysfunction) and to mortality. Further, for middle-aged people, independent associations have been shown for metabolic syndrome prevalence for time spent in SED (odds-ratio 2.38), in LPA (0.50), in MVPA (0.33), and aerobic fitness (0.24) [29].

Thus, daily increase in total-PA, LPA and MVPA and decreases in SED-total and SED-bouts have positive associations with numerous health outcomes and increased longevity in several longitudinal and cross-sectional accelerometer studies. Moreover, interventions promoting PA can reduce health- and social costs significantly, especially among older adults and those with poor health caused by physical inactivity [83,85,86]. Even small increases in PA give substantial health care cost savings and prevention of hospitalization [86]. In the present study, the exercise group succeeded in significantly improving several PA- and SED- parameters, emphasizing that this type of intervention may contribute to better health in different ways for older adults. Since large world-wide trends show that a substantial number of people spend too much time sedentary and are insufficiently active physically, policies to increase population levels of PA and reduce time spent in sedentary behavior need to be prioritized and up-scaled urgently.

### Strength and limitations

This is the first intervention study including two separate pre-tests performed with accelerometers in older adults showing improvements among several accelerometer-assessed parameters for both daily movement patterns (Total-PA, MVPA, LPA, MVPA-bouts) and sedentary behavior (SED-total, SED-bouts) in a supervised exercise intervention for community-dwelling relatively healthy older adults compared to a control group. Such previous interventions are absent. The more objectively assessment with accelerometers gives more valid estimate of actual PA- and SED-patterns than self-report methods do. Moreover, a strength is that we described results in both min/d and %-of-daily-wear-time, whereas most previous publications generally reported only one of these two units. For instance, the global recommendations for MVPA are expressed in minutes per week, and for this parameter, we found a significant improvement for the exercise group in the unit expressed in minutes.

Furthermore, the present data is compared to previous reports regarding supervised exercise interventions in older adults, sex and age groups. In addition, we present information about proven relationships between accelerometer-assessed parameters and various health outcomes for older adults in longitudinal and cross-sectional studies. Another strength is that the present supervised exercise sessions contained a combination of aerobic and resistance training, which in older adults seems to have a better effect than either training form alone, to counteract the health negative effects of SED behavior, including cardiovascular, mental and musculoskeletal functions [2]. Thus, the offered training sessions in the present study are in harmony with global physical activity guidelines for older adults, regarding including muscle-strengthening activities at moderate or greater intensity that involve all major muscle groups on at least two days a week and balance training [1,7,13].

A limitation is that the control group was not randomized in relation to the exercise group. The participants were recruited for the exercise intervention separately to the control group, a fact which may have affected the results. However, a control group has often not been included in previous supervised exercise interventions with accelerometer evaluations in relatively healthy community-dwelling older adults. The participants in the two groups in this study were rather comparable. They originated from the same suburb and all were relatively healthy and had a similar distribution of men and women. In addition, no significant differences in daily movement patterns were seen between the exercise group and control group at pre-tests, suggesting that there was no skewing between the groups at baseline level. Another limitation with accelerometer measures is that the type of SED behavior cannot be obtained with the present method, e.g., if the SED is stationary or sitting, neither if the SED is mentally active or mentally passive, which will influence mental health in different ways [87]. However, breaking up the SED-behavior in bouts compared to prolonged sitting is related to improved mental health with lower symptoms of depression and anxiety in adults [88]. In the present study we found significantly decreased time spent both in SED-total and in SED-bouts for the exercise group.

## Conclusions

The current study proposes good test-retest reliability of sensor-based physical activity and sedentary patterns in older adults prior to a supervised exercise intervention. In the group exercising twice weekly over eight weeks, we found no evidence for compensatory behaviors at the End-test, with decrease in any measured physical activity parameter or increase in time spent sedentary. The exercise- compared to the control-group significantly increased total-PA and MVPA (min/day), and decreased SED-total and SED-bouts (%-of-total-daily-wear-time), and SED-bouts (min/day). Thus, older adults participating in eight weeks of supervised physical activity twice/week improved several PA- and SED-parameters. The results from the present study indicate that not only MVPA should be assessed, but also total-PA and LPA, as well as total-SED-behavior and SED-bouts when evaluating daily movement patterns with accelerometers in older adults, since all these parameters could be affected differently depending on type of interventions performed and are all independently associated with different health outcomes.

These findings may contribute to valuable knowledge for future public health strategies to improve physical activity and sedentary behaviors and thereby health and quality of life in older adults. As the present-study was based on community-dwelling individuals, further studies are needed to investigate reliability in other populations. In addition, more studies of exercise intervention are needed to permit comparisons and follow-up measures to see any possible long-term effects.

## Supporting information

S1 Data (XLSX)
